# Cases of Thoracic Disease

**Published:** 1849-07

**Authors:** Thomas C. Osborne

**Affiliations:** Erie, Alabama


ART. III.—*Cases of Thoracic Disease.* By Dr. THOMAS C. OSBORNE, of Erie, Alabama.

CASE I. *Tubercular Phthisis—death from cerebral embarrassment.*—Patsy, a black woman, aged 27 years, is the girl noticed as the second in my cases of dropsy, published in this journal (2d series, vol. 7, page 238.)

After recovering from the ascites her health was apparently better than it had been at any time during the amenorrhea, but the menses never returned up to the period of dissolution. The most remarkable circumstance in her history, however, is the fact that she was never known to complain of cough or pectoral uneasiness, and, with the exception of the catamenial interruption, she was regarded as one of the most healthy among the field hands by her owner, who even attributed this defect to the action of the "polypody," or "cotton-root tea," which, it was believed, the negro women were in the habit of using to prevent child-bearing.

On the 13th of November, 1845, she was attacked with acute headache, attended with a furred tongue and obstinate constipation, for which I ordered an emetic of ipecac., to be followed at proper intervals, by purgative doses of calomel and jalap, until the bowels were sufficiently evacuated.

On the 15th, although still complaining, she was ordered to her work, and noticed no more until the 20th, when I was requested to ride into the field and see if anything ailed her; for it was thought she was inclined to dissemble and avoid labor in that way. She had then a vacant and troubled expression of the eyes; irregular and tremulous motion of the hands, dry, hot, and ashy skin; fiery tongue, with a heavy brown fur down its center; parched lips; thirst; frightened and broken pronunciation; hurried breathing; extremely feeble, quick, and frequent pulse; tenderness in the epigastrium; nausea; loss of appetite, and constipated bowels. She was sent to the house, bled, cupped over the temples and pit of the stomach, had a warm stimulating bath, and afterwards a sinapism over the spine and abdomen; took thirty grains of calomel, combined with five grains of ipecac., to be assisted in four hours, if necessary, by an ounce of castor oil.

21st. No better. Bowels freely opened by medicines, and dejections are of a bilious character. Pulse the same. Reapply cups and sinapisms.

22d. Became delirious last night, and while the nurse slept stripped off her clothes and laid down on the floor at the back part of the house, where she was found next morn ng cold and almost pulseless; with muttering delirium; subsultus tendinum; picking at the bed clothes; eyes filmy and thrown back. Frictions with hot mustard, pepper, and turpentine; blisters to extremities and scalp; calomel in ten grain doses every four hours; gruel enemata.

23d. Worse. No action on the bowels since the 21st; blisters have failed to vesicate; stupor and insensibility. She died about midnight.

*Necroscopy, twelve hours after death. Head.*—Meninges extremely vascular, and turgid with black blood. Brain natural. Lateral ventricles vascular, and contained an ounce or more of yellowish serum.

*Thorax.*—The principal seat of organic lesion was found in this cavity, where, in truth, it was least expected. The costal and pulmonary pleura, over the anterior and lateral parts of the right lung, were loosely adherent by several patches of unorganized lymph, while the posterior portion was united so tenaciously that it was impossible to separate the tunics with the edge of the knife without constant mutilation. The pleural surfaces were free posteriorly on the left lung, but there were anteriorly two spots of false membrane, an inch in diameter, of recent formation. The lungs were engorged, and when pressed a bloody froth issued from every point of the incision. Tubercles, of various sizes and in great abundance, were found in the superior lobes of both lungs; none of them, however, had the least appearance of softening. Heart normal.

*Abdomen.*—The stomach was stained with a single patch of the speckled discoloration, of diminutive size, near the pylorus. Liver and intestines healthy. Spleen enlarged and very firm. The omentum (especially the gastro-colic) and mesentery were excessively vascular, and turgid with red blood, so much so that the natural color of the tissue was almost obliterated. An immense number of miliary granulations were also found in them.

It is sufficiently proved that tubercle sometimes destroys its victim before the stage of softening is established, by inducing inflammation of the pulmonary tissue; but it is not equally clear that the presence of this heterologous formation in one part may kill the patient by exciting a fatal determination in a distant organ, as the foregoing case would seem to imply.

CASE II. *A case of Anemia, with the physical signs of heart disease.*

The subject of this case is a boy 8 years of age, with light hair, blue eyes, and cadaveric complexion. The anemia under which he is suffering dates back two years. It has been constantly growing upon him since that time, owing, no doubt, to the fact that he has been brought up in a low marshy district, which is inundated with every swell of the Warrior river, where he has for the last four years suffered repeated attacks of intermittent fever, followed by enlargement of the spleen, and looseness of the bowels. The tallow complexion, bloodless lips, swollen abdomen, and shrunken extremities, give him the appearance of a stealthy "dirt-eater," but I am assured that this is a practice from which he is entirely free.

December 18th, 1848. Called to-day: learned that during the month previous he had frequent and some very protracted fits of fainting, each followed by more or less severe palpitation, and that now it is impossible for him to walk across the floor without a return of them. There is no appetite; the bowels are sluggish; restlessness at night, and his sleep is disturbed by frightful dreams; mental apathy; foul tongue; offensive breath, and edematous condition of the inferior extremities.—Upon carefully exploring the case the following were the principal results obtained: High grade of fever; pulse 132, of inferior development, regular, equal, and synchronous with the ventricular contraction of the heart; thirst; tongue pale and furred; bowels inactive; hurried respiration, and occasional hacking cough. Percussion elicited nothing unusual. The first sound of the heart loud, audible at the distance of three feet, and communicating by its impulse a notable jar to the bedclothes; bellows sound heard near the sternum, intense and prolonged, occupying the second time and completely masking the auricular sound; respiratory murmur normal; deep inspiration excites cough, and uneasiness in the precordial region.

*Prescription.*—Dry cups over infra-scapular region; sinapisms on front of chest; hot foot-bath.

℞. Calomel grs. ii.
Ipecac. gr. i.—M.; to be repeated every three hours.

20th. Better. Medicines have been regularly administered, and free purging of fecal and bilious matter followed the fourth dose. Skin slightly moist; fever diminished; pulse 110; headache relieved; sounds and impulse of the heart decreasing in intensity; some appetite. Give the medicines every five hours, and one hour after each dose a wineglassful of the following mixture: hyd. potass., grs. xvi; citrat. ferri, grs. xxxii; aqua font, Oi. Mix.

22d. Improving. Give midicines three times daily; nourishing diet.

30th. Convalescent. Sounds of heart normal. Respiration easy. Appetite good. Omit former medicines, and give mur. tinct. ferri, three times daily. Dismissed.

Case III. *Acute Pleuritis.*—At the date of the following notice, Influenza prevailed rather epidemically in this vicinity; many of the cases of which disease, being neglected, ran into a high grade of inflammation in the pulmonary apparatus, as this and the subsequent examples will partially attest.

January 3, 1849. Called to prescribe for Miss P., aged 16 years, who has, for several days, had the prevailing influenza, and was taken this evening, after exposure, with violent pain in the left side, dyspnea, etc. Saw her three hours subsequent to the attack: pulse 120, full and hard; considerable fever; hurried and difficult respiration; pain in the left mammary region; distressing cough, with copious expectoration of thick, tenacious mucus, which had existed three or four days; tongue clean, and bowels regular. On applying the stethoscope over the seat of the pain, a sound of friction came up from a surface of about two inches in diameter; respiratory murmur on same side rather distinct; on the right side there was a subcrepitant rhonchus at the summit, which gradually diminished as the exploration was carried towards the base of the right lung. Ordered venesection ad deliquium animi. The patient replaced in bed, and a large sinapism applied over the left side, after which she took one hundred drops of laudanum in a glass of lemonade.

4th. Rested well all night; felt no return of the pain since the bleeding; thinks she is quite as well as before the attack.

CASE IV. *Influenza.*—D. L. McCoy, carriage maker, aged about 30 years, was taken with chilly sensations and shivering, followed by high fever, on the 31st of January, 1849. Saw him on the 2d of February. There is acute headache, redness of the eyes, with intolerance of light and sound, pain in the back, pains and soreness in the chest, dyspnea, cough, with expectoration of thin acrid mucus, sneezing, coryza, thirst, hoarseness, sore-throat, red tongue, costiveness, and a pulse beating one hundred, full and bounding.

*Prescription.*—Venesection 30 ounces.

℞. Syr. tolu.,
Paregoric, ää ℥i,
Vin. ant., ℥ss;

Dose—A tablespoonful every hour.

Cooke's pills at bedtime, immediately after rising from tepid bath.

3d. At work; feels well except weakness; pills acted finely.

CASE V. *Hemoptysis—Tertian Fever.*—January 14th, 1849, called to John, a mulatto, aged 18 years, short, thick stature; has had several severe attacks of febrile disease in the course of the last few years, and is extremely liable to 'take cold;' had, occasionally, hacking cough. While lifting a heavy weight, on the 10th inst., complained of feeling, on a sudden, as if 'something broke loose in the right side.' A chill, succeeded by high fever, cough, and bloody expectoration came on soon afterwards, which passed off in the next twelve hours with free perspiration. On the 13th, another chill with greatly aggravated fever, etc. Condition now as follows: hot and dry surface; thirst; red and fissured tongue; tenderness in epigastrium; pulse 90, full and compressible; cough every few minutes; expectoration consists entirely of florid blood, which is brought up easily and in considerable quantities.

*Physical signs.*—No dullness on percussion; subcrepitant râle, with large bubbles well personated at summit anteriorly of right lung, which gradually diminishes as the base is approached; posterior portion, respiratory murmur rather strong. In the left lung the normal murmur was unembarrassed.

*Treatment.*—Venesection 25 ounces. Sinapisms to pit of stomach.

℞. Nitrat. potassa, ℥i;
Mucilage slippery elm, Oi;
M. Dose—a tablespoonful every three hours.

Cooke's pills at bedtime, and quinine as soon as sweating appears.

15th. About as yesterday; feels very weak and faint. Pills brought away several bilious discharges. Complains more of pain at pit of the stomach; continues to cough up blood freely; has not sweated. Apply wet cups to epigastrium, and follow them with another mustard poultice. Continue the nitrate of potash, and give five grains of sulphate of quinine every two hours until he has taken six doses.

16th. Much better. Skin moist and pleasant; commenced sweating last night; cough less troublesome, and expectoration merely streaked with blood. Subcrepitant rhonchus persists, but is much less noisy. Continue nitre, and give quinine again to-morrow.

Case VI. *Broncho-pleuro—Pneumonitis.*—Jackson, a mulatto, aged 23 years, with rather scrofulous diathesis, had chills some two or three weeks since, from which he was slowly recovering when attacked with influenza, which, however, did not seriously incapacitate him until last night, (March 25, 1849,) when he was suddenly seized with a sensation of stricture across the chest, distressing dyspnea, high fever, pain in the frontal region, aggravated by almost continual cough, which was attended with copious expectoration of thick, yellowish, tenacious mucus; retching, and lancinating pain seated near the left mammary region.

Saw him on the morning of the 26th, four hours subsequent to the attack. Pulse 90, full and hard; respiration 30, and jerking; hot and dry skin; thirst; furred tongue, and constipation of the bowels; mucous rhonchus abundant and present over all the anterior portions of both lungs, but strongest on superior lobes; posteriorly puerile respiration; sound of friction on a small space outside of left nipple; vesicular murmur superficial on the lateral portion of the right lung, between the third and sixth ribs, occupying a surface of about three or four inches in diameter. Percussion elicited nothing unusual.

*Treatment.*—Venesection to 35 ounces. Cups to eight ounces from right side, and afterwards apply sinapisms over front of chest.

℞. Hyd. chlorid. mitis, grs.x.
Pulv. ipecac., grs.viii.
Pulv. opii, grs.ii.
M. et divid. in pil. x.

Give one pill every two hours.

4 o'clock, P.M. Feels better. Pulse 84; respiration 25. Has slept, and is sweating freely. Physical signs the same, except the friction sound, which is absent. No action on bowels.

*Prescription.*—Continue pills, and give alternately with them a tablespoonful of the following syrup:

℞. Tart. potas. et antim., grs.vi.

Tinct. scillæ, ℥ii.

Tinct. camph. c. ℥i.

Syrup. tolu, ℥viii. M.

Tepid pediluvium. Gruel enemata until the bowels have acted.

27th—8 o'clock, A.M. Worse. Exacerbation of fever came on after midnight; pulse 96; respiration 33; bowels relieved. Mucous râle heard only at summit of left lung anteriorly; condition of right lung persistent. Treatment the same.

5 o'clock, P. M. Pulse 106; respiration 33; thirst; physical signs same as last noticed. Ordered 10 ounces of blood to be taken from the right side by cups, to be repeated in five or six hours, followed by application of mush poultice. Saline aperient; tepid foot bath; mucilaginous drinks. Continue medicines.

28th—10 A. M. Passed a restless night; pulse 104; respiration 28; tongue red; several bilious evacuations since last visit. Normal murmur established in left lung: absence of crepitating râle, and, in its place, a tubular whiff; percussion dull at that point in right lung.

*Treatment.*—Venesection 12 ounces, to fainting. Sinapism to right side, to be followed by repeated mush poultices. Continue medicines.

6 o'clock, P.M. Pulse 90; respiration 25; cough and expectoration unchanged. He has not complained of pain since the first bleeding, except such as was occasioned in the frontal region by cough. The tubular whiff still continues. Ordered a blister over right lung. Continue the medicines as before.

29th. Passed a comfortable night; says he feels better. Pulse 84; respiration 27; tongue cleaning off; still sweats freely. Crepitant rhonchus heard in subclavicular and axillary regions of left lung. Prescribed a sinapism over left lung. Continue medicines.

30th. Pulse 112; respiration 29; blister healed. Physical signs same, except a slight improvement in subclavicular region of left lung. Warm bath; reapply blister; saline aperient. Continue medicines.

31st. Feels better. Pulse 100; respiration 28; urine clear and free; appetite good; slight salivation; says the warm bath relieved him more than anything that had been done. Crepitant rhonchus disappearing in left, and some improvement in condition of right lung. Continue syrup and ipecac., omitting calomel.

April 1st. Some nausea and vomiting of bile; fever; perspiration; pulse 96; respiration 35; puerile respiration in left lung, and sonorous râle and bronchophony in superior portion of right lung; ptyalism disappeared. Continue treatment, adding blue mass to ipecac.

2d. Pulse 88; respiration 36. Continue treatment, and give five grains sulph. quinine every three hours.

3d. Condition unchanged; continue treatment.

4th. Worse. High fever; stomach rejected quinine; pulse 96 and feeble; respiration 43; subcrepitant râle loud and noisy, in both acts of respiration, in right clavicular region, and sonorous rhonchus beneath the nipple. Mucous râle also in superior left lung. Ordered a blister over both lungs anteriorly; omit quinine; continue other medicines.

5th. Consultation with Dr. Jennings. Pulse 96; respiration 36, and more thoracic, mouth sore; physical signs nearly the same. Omit syrup, and give brown mixture, with teaspoonful doses of citrated solution of carb. ammon.

6th. Pulse 84; respiration 33.

℞. Syrup. senegæ, ℥viii.
Syrup. scillæ,
Tinct. camph. c. āā ℥i. M.

Dose—a tablespoonful every two hours, adding the solution of ammon. carb.

7th. Pulse 100; respiration 32; mucous râle disappearing, and bronchial respiration more abundant in both lungs. Continue treatment.

8th. Pulse 80; respiration 32; bronchial respiration extending; fever diminished.

9th. Pulse 96; respiration 23; bronchial respiration loud and apparently cavernous.

11th. Improving. Pulse 82; respiration 26.

14th. Continues improving. Pulse 88; respiration 26. Omit pills, and continue syrup.

20th. Physical signs of bronchial dilatation greatly diminished in intensity and area. Convalescence fully established.

Some of the cases of neglected influenza terminating in pneumonitis were so destitute of the characteristic symptoms that the real nature of the disease was not suspected, until post-mortem inspection revealed the distinguishing lesions. Of such a kind is the following instance, which, I am assured by the attending physician, was so insidious in its advances that, until forty-eight hours prior to dissolution, the patient continued to go about, with very little appearance of pulmonary suffering.

Case VII. *Necroscopy, thirteen hours after death.*—March 24, 1849, permitted to inspect the body of Peggy, a black woman, aged about 50 years.

Muscular rigidity incomplete; entirely absent in neck; some degree of animal heat remaining on the anterior and posterior portions opposite the heart. Abdominal fulness, but emaciation of the rest of the cadaver. On separating the flaps, made in the ventral parietes, the enormous dimensions of the liver arrested attention; it extended below the umbilical and filled up the hypochondriac regions. It was of a greyish color and very firm, almost grating under the knife. Gall bladder contained a small quantity of black bile.

*Stomach.*—Near the cardiac aperture, on the mucous surface, there was a patch of purple discoloration an inch in diameter; several smaller patches with considerable intervals were disposed over the great curvature. At the pyloric extremity were many small spots and patches of the diffuse and dot-like stain, of intense redness. Spleen and intestines healthy.

*Thorax.*—Anteriorly on both lungs the pleura were loosely united by several diminutive adhesions,—posteriorly the adhesion was tenacious and complete from base to summit. Heart normal. Lungs.—Gray hepatization in both; more extensive in the right; on scraping an incised surface, purulent matter exuded from minute points. About the roots and summit of both the lungs were crepitous, but in the latter parts there was quite a number of miliary granulations.

May 20, 1849.

